# Recent developments in the *CCP-EM* software suite

**DOI:** 10.1107/S2059798317007859

**Published:** 2017-05-31

**Authors:** Tom Burnley, Colin M. Palmer, Martyn Winn

**Affiliations:** a Scientific Computing Department, Science and Technology Facilities Council, Research Complex at Harwell, Didcot OX11 0FA, England

**Keywords:** Collaborative Computational Project for Electron cryo-Microscopy, *CCP-EM*, cryo-EM

## Abstract

As part of its remit to provide computational support to the cryo-EM community, the Collaborative Computational Project for Electron cryo-Microscopy (CCP-EM) has produced a software framework which enables easy access to a range of programs and utilities. The resulting software suite incorporates contributions from different collaborators by encapsulating them in Python task wrappers, which are then made accessible *via* a user-friendly graphical user interface as well as a command-line interface suitable for scripting. The framework includes tools for project and data management. An overview of the design of the framework is given, together with a survey of the functionality at different levels. The current *CCP-EM* suite has particular strength in the building and refinement of atomic models into cryo-EM reconstructions, which is described in detail.

## Abstract 2

An overview is given of the new *CCP-EM* software suite, including the underpinning framework, the current functionality and the distribution procedure. This first version of the suite has a focus on building and refining atomic models.

## 1. Introduction   

The Collaborative Computational Project for Electron cryo-Microscopy (CCP-EM) was initiated in 2012 to support the computational needs of the macromolecular electron cryo-microscopy (cryo-EM) community. To this end, it aims to support both software developers and users in a manner analogous to the way in which the long-running Collaborative Computational Project, Number 4 (CCP4; Winn *et al.*, 2011 ▸) has supported the macromolecular crystallography (MX) community. CCP-EM is mandated to provide user training and developer support and to establish a coherent community for the exchange of best practices and novel ideas.

The creation of the CCP-EM project has been described previously (Wood *et al.*, 2015 ▸). In this contribution, we specifically address the *CCP-EM* software suite: a multi-platform suite of tools that, in time, aims to cover all aspects of cryo-EM data processing from image manipulation to the building of atomic models, and to cover multiple techniques such as single-particle reconstruction (SPR), tomography and diffraction. Packaging tools together allows better management of structural biology projects, as well as better distribution and testing of software, to the benefit of both users and developers. Here, we describe the development of the *CCP-EM* software suite and its initial functionality.

The *CCP-EM* software suite was conceived as a generic framework that could support a wide variety of functionalities, whether written by ourselves or provided by external programs. As a collaborative project, the ability to incorporate programs from external partners is a high priority. There are clear conceptual similarities to other frameworks such as *Appion* (Lander *et al.*, 2009 ▸), *Scipion* (de la Rosa-Trevín *et al.*, 2016 ▸) and *Focus* (Biyani *et al.*, 2017 ▸). One unique feature of the *CCP-EM* framework is its close connection to the highly successful *CCP*4 suite for macromolecular crystallography, which arises out of historic links. This connection allows the reuse of software-engineering technologies deployed previously for *CCP*4, and the easy incorporation of crystallographic programs for the interpretation of high-resolution reconstructions. Nevertheless, the *CCP-EM* suite is distinct from *CCP*4 and is firmly directed towards the cryo-EM community.

A public beta release of the *CCP-EM* suite was made in 2016, and has been used since then in several CCP-EM training courses. The initial focus has been on fitting, building and refining of atomic models into high- or medium-resolution single-particle reconstructions. This focus partly reflects our historical links to the macromolecular crystallography community, but is also timely given the recent ‘resolution revolution’ (Kühlbrandt, 2014 ▸). The *CCP-EM* suite aims to assist microscopists who are perhaps obtaining high-resolution structures for the first time, and may be unfamiliar with topics such as reciprocal-space refinement or the use of structural restraints. Equally, for the many crystallographers who are moving into the cryo-EM field, the suite aims to help them adapt familiar tools to new data sets. In all cases, the *CCP-EM* suite provides convenient pipelining and data-management tools, which are becoming essential as cryo-EM moves to become a high-throughput and widespread technique (Stuart *et al.*, 2016 ▸).

In the following section, we describe the generic software framework and the design decisions that have been made. We cover the underlying software libraries which support applications visible to the general user, as well as facilitating further development. We then go on to give an overview of the current functionality, including small utilities and major programs. We finish with a quick discussion of future plans.

## 2. Software framework   

The *CCP-EM* software framework is primarily written in Python. Python is an interpreted language that is widely used in the scientific community; examples in structural biology include *CCP*4 (Winn *et al.*, 2011 ▸), *PHENIX* (Adams *et al.*, 2010 ▸), *PyMOL* (Schrödinger), *Scipion* (de la Rosa-Trevín *et al.*, 2016 ▸) and *DIALS* (Waterman *et al.*, 2016 ▸). Its convenience and shallow learning curve have aided its popularity, and furthermore it is cross-platform as it does not require compilation.

### 2.1. Libraries and utilities   

**Figure 1:**
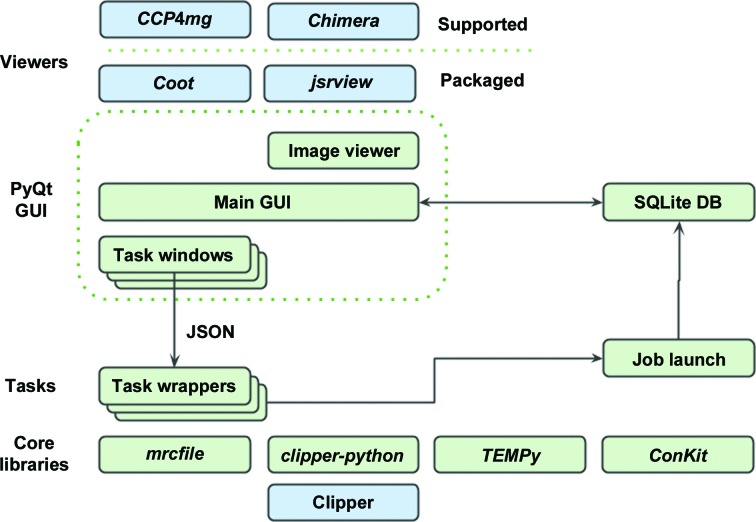
Architecture of the *CCP-EM* software suite. The task wrappers and core libraries shown in green are written in pure Python, whereas the GUI layer is written in PyQt4. The GUI thread is independent of the job processes; task progress is monitored by a job-launch module and is recorded in an SQLite database. JSON files serve as intermediaries allowing the task to be controlled ‘headless’ without the GUI layer.

The *CCP-EM* software framework has a modular organization which can be roughly divided into three layers (Fig. 1 ▸). The top-level GUI (graphical user interface) layer is written using the PyQt toolkit. This provides a simple graphical interface to the associated programs. Distinct from this is the mid-level management layer, which is written in pure Python. This provides a bridge between the GUI layer and the third layer: the set of functional programs. These programs originate from collaborating developers and are written in a wide variety of languages (including C, C++, Fortran and Python) with distinct control methods and input conventions.

**Figure 2:**
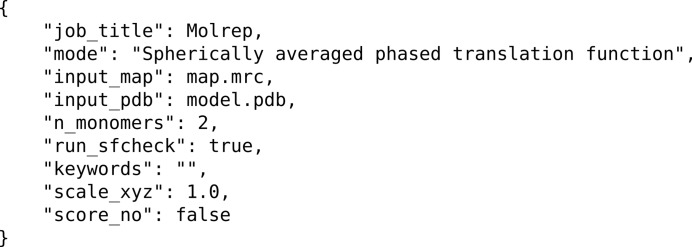
JSON files are used as a convenient, human-readable store of parameters and provide a consistent input for *CCP-EM*-supported applications. In this example, input parameters for a *MOLREP* job are shown, including the use of a spherically averaged phase translation function and searching for two copies of the search model in the EM volume.

The second, management layer provides Python task wrappers for each of the functional programs and gives a common interface style accessible *via* the GUI or the CLI (command-line interface). Command arguments are in the JSON (JavaScript Object Notation) format. This is a lightweight metadata format which is commonly used as it is more human-readable than other markup formats (for example XML) and is fully supported in the Python standard library (Fig. 2 ▸ shows an example JSON input file). Each specific task is derived from from two principal base classes. A CCPEMTask class provides the pure Python wrapper to the application, defining in a generic way the parameters appropriate to the task, which are then translated as inputs to the various APIs of the underlying applications. Workflows can be constructed using these wrappers, allowing tasks to be linked together and/or run in parallel. The second base class, CCPEMWindow, contains the PyQt4 functionality that provides the GUI window. Each CCPEMWindow holds an instance of the relevant CCPEMTask to allow access to the defined input arguments and trigger the activation of a job. A simple PyQt image viewer has been developed to visualize the contents of MRC-format image stacks.

The software framework has a suite of unit tests to ensure the reliability and reusability of the codebase, and the support programs also have a series of implementation tests to allow autonomous testing of the suite before distribution. It should be noted that the suite has a number of third-party dependencies. These have been selected with care, and are mainstream and well maintained. Every effort has been made to ensure the modularity of the framework such that in the event of a dependency becoming unavailable or unsuitable it could be substituted with an equivalent, either sourced from another third party or developed in-house.

### 2.2. Python MRC file library   

The MRC file format is one of the principal formats for cryo-EM data, and is used in common programs such as *RELION* (Scheres *et al.*, 2008 ▸) and for the deposition of experimental volumes in the EMDB (Tagari *et al.*, 2002 ▸; Lawson *et al.*, 2016 ▸). Closely related to the CCP4 map format, it can be used to store individual micrographs, stacks of two-dimensional images, three-dimensional volumes and stacks of three-dimensional volumes. Several variants of the MRC format had emerged, but recently the developers of several major EM software packages agreed a standardized definition, known as the MRC2014 format (Cheng *et al.*, 2015 ▸), together with a process for agreeing future revisions. In order to allow developers to use MRC files as easily and flexibly as possible, we have written *mrcfile.py*, which is an open-source, stand-alone Python library for the reading, writing and validation of MRC2014 files. It is available in the *ccpem-python* environment (see §[Sec sec2.3]2.3), but can also be obtained separately from PyPI (https://pypi.python.org/pypi/mrcfile) or GitHub (https://github.com/ccpem/mrcfile).

**Figure 3:**
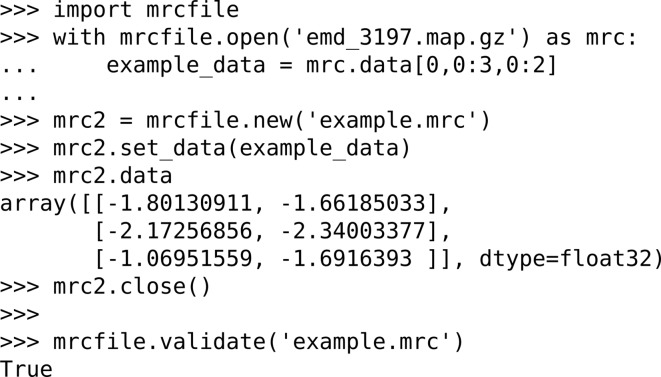
Basic usage of the *mrcfile* Python library. In this example, a compressed map downloaded from the EMDB is opened and a 2 × 3 slice of data is taken from it. A new MRC file is then created, the data are copied into it and checked, and the file is closed. Finally, the file is validated to confirm that it complies with the MRC2014 standard.

The main design goals of the *mrcfile* Python library are to make data from an MRC file available as a *NumPy* array (van der Walt *et al.*, 2011 ▸) *via* a clear and simple interface, and to allow easy validation of MRC files against the MRC2014 standard. Python’s standard file-handling semantics are used as far as possible; for example, MRC files are opened by calling the mrcfile.open() function and closed after use by calling close(). The file header and data arrays are simply accessed *via* header and data fields on the open MRC file object. Files can be validated for compliance with the MRC2014 standard using the mrcfile.validate() function. Other features include seamless support of gzip-compressed files (as used for maps downloaded from the EMDB) and a memory-mapped file option for fast random access to small chunks of very large files. To make it as simple as possible to install and use, *mrcfile* is written in pure Python (fully compatible with Python versions 2 and 3) and its only dependency is *NumPy*. A brief example of its usage is shown in Fig. 3 ▸. A full usage guide and a description of the underlying design are available in the online documentation (http://mrcfile.readthedocs.org/).

### 2.3. Python toolkit for EM   

A number of other Python modules are available within the *CCP-EM* software framework. These are used internally in the suite, but may also act as a useful toolkit for programmers wishing to write their own scripts. Currently, Python 2.7.11 is packaged with the suite along with specific libraries developed by CCP-EM and collaborators and additional open-source dependencies. The latter include common scientific modules such as *NumPy* (van der Walt *et al.*, 2011 ▸), *SciPy* (Jones *et al.*, 2001 ▸), *Biopython* (Cock *et al.*, 2009 ▸) and the Python imaging library *Pillow* (https://python-pillow.org). This Python ‘ecosystem’ can be accessed by invoking *ccpem-python* from the command line.

Led by the University of York, *clipper-python* has been developed to provide Python bindings to the established C++ Clipper library (Cowtan, 2003 ▸), which underpins a number of *CCP*4 programs such as *Buccaneer* (Cowtan, 2006 ▸) and which is also used in *Coot* (Emsley *et al.*, 2010 ▸). The Clipper library was originally developed to aid the organization of crystallo­graphic data and enhance the performance of crystallographic computation, and as such has many features that are applicable to EM data processing, in particular for high-resolution model building. Of particular relevance is the NXmap class, which is a noncrystallographic map class that stores a map of an arbitrary data type. In contrast to the Xmap crystallo­graphic map class, it is finite in extent and has no symmetry, and is therefore appropriate for EM volumes derived from SPR or tomography (Cowtan, 2003 ▸). *clipper-python* exposes many of the C++ arrays as pythonic *NumPy* arrays; this in turn conveniently links the specific objects found in MX and EM (such as map volumes) to the *NumPy* library for the rapid development and deployment of new algorithms.

*ConKit* (Simkovic *et al.*, 2017 ▸), developed at the University of Liverpool, is a Python interface for the analysis, manipulation and visualization of evolutionary contact predictions from several alternative algorithms: *HHblits* (Remmert *et al.*, 2012 ▸), *JackHMMER* (Johnson *et al.*, 2010 ▸), *HHfilter* (Remmert *et al.*, 2012 ▸), *CCMpred* (Seemayer *et al.*, 2014 ▸), *PSICOV* (Jones *et al.*, 2012 ▸) and *bbcontacts* (Andreani & Söding, 2015 ▸). This library facilitates the inclusion of additional structure restraints inferred from deep-sequencing data, based on contact predictions made by external programs and provided in one of a number of data formats. Initial efforts using this approach for guiding models into cryo-EM density have proved successful (Schep *et al.*, 2016 ▸).

## 3. Graphical user interface and job management   

**Figure 4:**
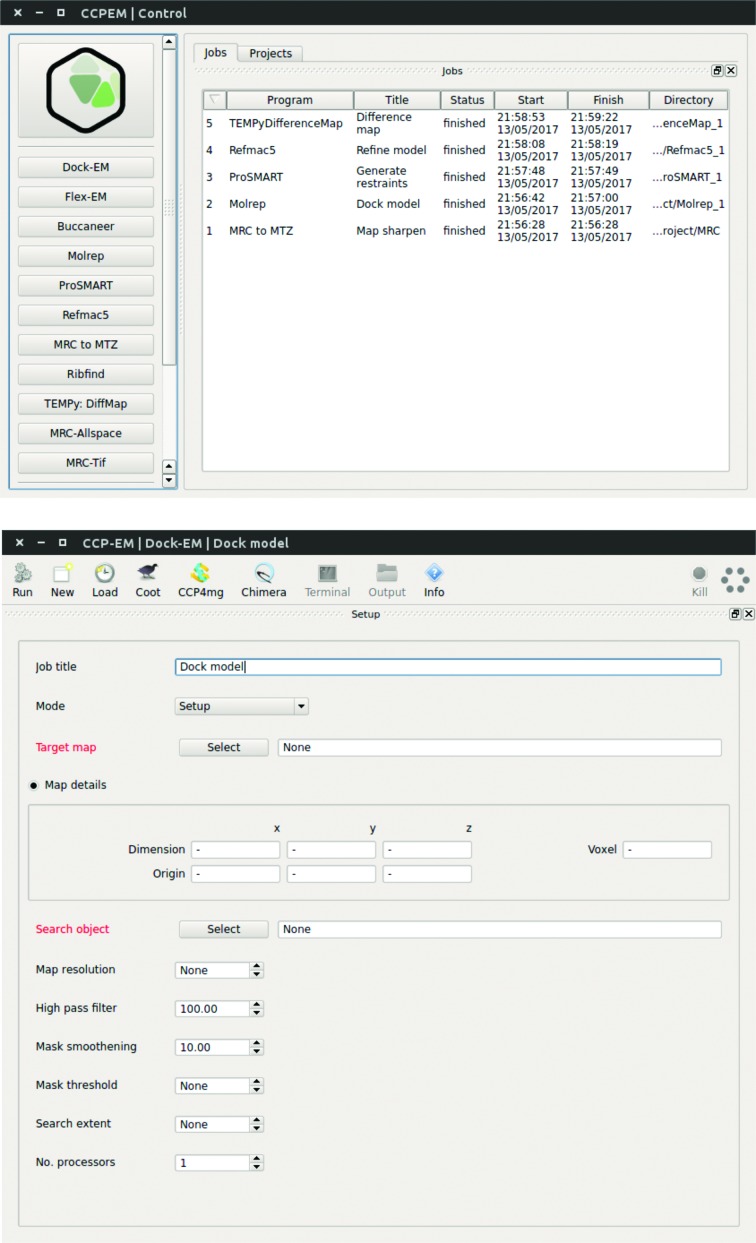
*CCP-EM* project and task window. Top: *CCP-EM* project window showing the taskbar which is used to launch applications on the left and the project job history on the right. Bottom: example of the *CCP-EM* *DockEM* task. The toolbar at the top gives rapid access to molecular-graphics programs, job files, documentation and job launch. The input parameter setup tab is shown below, with required inputs highlighted in red. Additional launcher and results tabs appear as the job is launched and completed, respectively.

Initial interfaces have been provided for a series of model-building tools applicable to high-to-medium-resolution (<10 Å) volumes. These tasks can be accessed *via* the control GUI, as stand­alone task GUIs or *via* the CLI. The control GUI (Fig. 4 ▸) contains simple project-management utilities allowing users to create new projects, record a chronological list of jobs and monitor the status of ongoing processes. Details of projects and jobs are stored in an SQLite database that is integrated *via* PyQt4/Qt4 bindings. The GUI is designed such that jobs are launched as detached, separate processes, so that the main GUI thread can be launched and terminated without interfering with long-running subtasks that are launched from it.

Each new project created by a user is stored in a separate directory, and child tasks of that project are stored in individual subdirectories. The top project directory stores the SQLite database file used to record the following information for each task: incremental job number, date and time of job initiation and completion, task type, task name, job location and current status. Clicking on the task entry launches its task window. The left-hand toolbar launches a new instance of the selected task. If Test mode is selected, new tasks will be preloaded with parameters and data from that task’s unit test to allow new users to trial an application and examine the expected output.

Each task window has a similar basic layout (Fig. 4 ▸), with a toolbar and four main tabs: Setup, Pipeline, Launcher and Results. The toolbar provides a Run button for launching the task, a New button for cloning the current task (*i.e.* preserving any defined inputs) and a Load button for opening previous runs. The Coot, CCP4mg and Chimera buttons provide quick links to commonly used molecular-graphics programs. The Terminal and Output buttons launch a terminal or file-browser instance in the task’s subdirectory to allow rapid file navigation, whilst the Info button displays a brief description of the task and provides clickable hyperlinks to the task’s online documentation. Finally, there is a Kill button for terminating the task and a green hexagon status indicator. The status indicator is grey when ready, spins when running and is coloured green on successful completion of the task or red upon failure.

The Setup tab allows input parameters to be entered by the user. Appropriate defaults are used wherever possible and required user input is highlighted in red. Each input has a tooltip, which is visible upon mouse hover and gives a brief description to aid new users. Programs originally developed for the *CCP*4 suite have keyworded input for specifying extended functionality where appropriate.

The Pipeline tab shows the individual jobs that make up the task. For example, in the *MOLREP* task three processes are launched in series. Initially *SFCHECK* (Vaguine *et al.*, 1999 ▸) analyzes the input .mrc file, followed by the main *MOLREP* (Vagin & Teplyakov, 2010 ▸) process and finally a third *SFCHECK* process comparing the fitted structure with the input map. The status of each subprocess is colour-coded: grey for ready, blue for running and green for finished. Clicking on each job displays information in the right-hand widget, including the job’s log file. Double-clicking on the log file opens the text file in the user’s standard editor. If the standard error file of the job process is greater than 0 bytes in size then it is also displayed to alert the user to potential problems.

The Launcher tab highlights important files associated with the task, for example in the *REFMAC* (Murshudov *et al.*, 2011 ▸) refinement task the input PDB and map files are shown along with the refined output PDB file. A brief description of each file is given and double-clicking will launch an appropriate application to view the file. For standard files (for example text or PDF files) the user’s normal desktop application will be launched, while for structural biology files (*e.g.* coordinate files or map volumes) the user can select their preferred molecular-graphics (MG) program from a list of those available. Clicking Open Selected will open all selected files at once, allowing the rapid visualization of the results of a task.

Molecular-graphics integration is provided for three of the most common MG programs: *Coot* (Emsley *et al.*, 2010 ▸), *Chimera* (Pettersen *et al.*, 2004 ▸) and *CCP*4*mg* (McNicholas *et al.*, 2011 ▸). *Coot* is packaged with *CCP-EM*, while the others are used if available. (*CCP-EM* searches the system paths to find the expected MG executable, or users can explicitly set this path *via* the *CCP-EM* settings.) In the simplest cases, tasks will launch the MG program and the selected files will be automatically loaded. However, for some tasks specialized run scripts have been produced. For example, the *MRC to MTZ* task loads all calculated map coefficients in *Coot* so that different degrees of sharpening and blurring can be compared (see §[Sec sec4.8]4.8), the *TEMPy:DiffMap* task loads scaled maps into *Chimera* and the *DockEM* task allows selections of best hits to be displayed in *Chimera*.

Finally, the Results tab uses a PyQt Webkit widget to display an HTML file of the task’s results. This HTML file is produced by the *jsrview* package from *CCP*4 (Winn *et al.*, 2011 ▸) *via* its Python bindings *pyrvapi* (log files from *CCP*4-derived programs are pre-processed using the *CCP*4 smartie library; Briggs, 2007 ▸). The *jsrview* package was initially developed by E. Krissinel to support dynamic HTML output in *CCP*4’s *jsPISA* webserver (Krissinel, 2015 ▸). This package allows a developer to create dynamic and interactive HTML pages using a library of high-level C functions featuring various graphical widgets (such as plots, molecular graphics, tables, buttons, comboboxes *etc.*) and nested layouts (tabs, folders and grids). The functions generate a task file with a pseudo-program for a real-time JavaScript interpreter, based on jQuery, which is loaded in the browser widget using a bootstrap HTML page. The package may be used in programs written in C, C++ and Fortran, as well as Python through the set of corresponding bindings. The resulting output may be served either from the local file system or *via* a remote server. This would allow *CCP-EM* to efficiently transfer to web-based applications in the future if required.

## 4. *CCP-EM* tasks   

**Figure 5:**
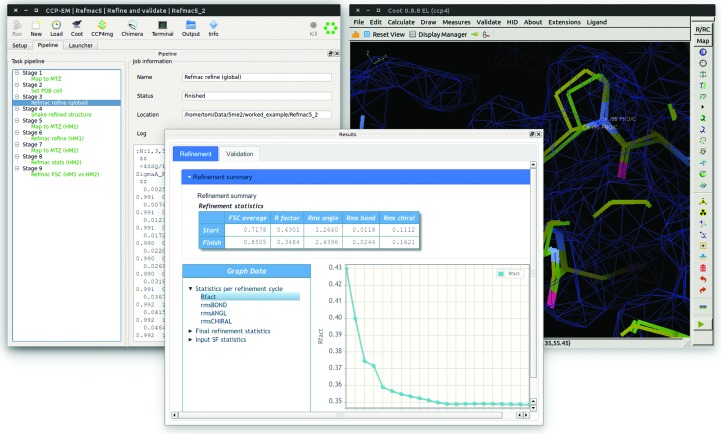
*CCP-EM* *REFMAC* task for the refinement and validation of atomic models in high-resolution cryo-EM maps. The single task includes the generation of structure factors from an input reconstruction, as well as the application of multiple blurring and sharpening factors. The left panel shows the *CCP-EM* pipeline for refinement and validation against experimental half-maps. The centre panel shows the results tab and the right panel shows the input and refined model in *Coot*.

**Figure 6:**
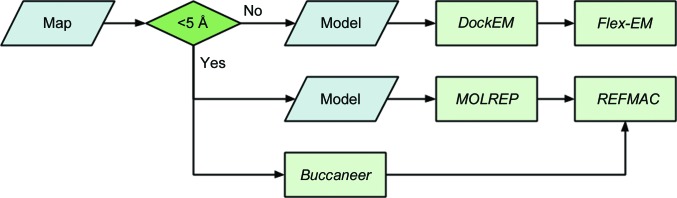
Model-building pipeline in *CCP-EM*. For maps (or segments thereof) with resolutions of less than ∼5 Å and an appropriate model it is suggested to try *DockEM* followed by *Flex-EM*. For higher resolution data *MOLREP* and *REFMAC* can be used for refinement if a suitable model is available. If no model is available then *Buccaneer* can be used to build a model *de novo*. Note that for medium-resolution data sets a combination of approaches is recommended.

The initial set of applications in the *CCP-EM* suite is focused primarily on model building into volumes derived from single-particle reconstruction and high-resolution subtomogram averaging. Fig. 5 ▸ shows in detail the task for refinement of atomic models, while Fig. 6 ▸ gives an overview of possible routes through the suite.

If a user has an appropriate atomic model available, such as a homologous domain from a high-resolution crystal structure, then either *MOLREP* (Vagin & Teplyakov, 2010 ▸) or *DockEM* (Roseman, 2000 ▸) can be used to dock the structure into the cryo-EM volume. If no atomic model is available then *Buccaneer* (Cowtan, 2006 ▸) can be used for *de novo* model building. The next step is to refine the structure, *i.e.* to optimize the fit of the atomic model to both the experimental volume and the established stereochemical restraints. Either *Flex-EM* (Topf *et al.*, 2008 ▸) or *REFMAC* (Murshudov *et al.*, 2011 ▸) can be employed here. Initially developed for low-resolution crystallography, additional structural restraints can be helpful when the information content in the map density is low. *ProSMART* (Nicholls *et al.*, 2012 ▸) can used to generate such additional restraints for *REFMAC*. *Flex-EM* requires rigid-body definitions and these can be produced using the helper program *RIBFIND* (Pandurangan & Topf, 2012*a* ▸,*b* ▸). *CCP-EM* also includes the *TEMPy* library (Farabella *et al.*, 2015 ▸), and task interfaces for *TEMPy:DiffMap* (difference map) and *TEMPy:SMOC* (Segment-based Manders’ Overlap Coefficient) for structural validation are provided.

Other general utility tasks that are provided include *MRC to MTZ* for the conversion of map files to structure factors, and *MRCEdit* for viewing and editing MRC header information. *CCP-EM* also maintains the MRC image-processing system (Crowther *et al.*, 1996 ▸), and GUIs are provided for the *mrc*2*tif* and *mrcallspacea* routines.

### 4.1. *MOLREP*   

*MOLREP* (Vagin & Teplyakov, 2010 ▸) was originally developed as an automated program for molecular replacement in *CCP*4 and has since been adapted for rigid-body docking into cryo-EM maps. It was extensively used by Amunts *et al.* (2014 ▸) for the placement of homologous structures into the yeast mitochondrial large ribosomal subunit and other large complexes (for further methodological details, see Brown *et al.*, 2015 ▸). *MOLREP* works in reciprocal space, is relatively fast and is best suited to high-resolution maps. Although by default the single best-fit structure is returned, multiple solutions can be examined by specifying the number of copies expected in the cryo-EM volume. *MOLREP* uses a rotation function with a phased translation function. A spherically averaged phased translation function can also be used in which the centre of mass of the input model is found before optimizing the orientation (Vagin & Isupov, 2001 ▸), which can be advantageous when using distantly related search models or with lower resolution maps.

### 4.2. *DockEM*   

*DockEM* (Roseman, 2000 ▸) is an exhaustive rigid-body docking algorithm. With a defined angular sweep, all possible orientations of the search model are examined within a defined area of the target map and the cross-correlation (CC) score is calculated. The top ten best poses are returned (as ranked by CC) and can be selected to be viewed in *Chimera*. Once the *DockEM* scoring has been completed, the analysis can be repeated to return more poses and/or the peak radius, which defines the exclusion threshold for neighbouring solutions, can be altered.

Owing to the exhaustive nature of *DockEM* it is slower than *MOLREP*, but it is useful for low-resolution data sets where *MOLREP* cannot produce an unambiguous solution. The scoring function, along with visualization in *Chimera*, allows users to consider the relative quality of multiple possible fits.

### 4.3. *Buccaneer*   

*Buccaneer* (Cowtan, 2006 ▸) is used for automated *de novo* model building and originates from the *CCP*4 suite. Required inputs are an EM map (MRC format) and an expected sequence (FASTA format or similar). Partial PDB models can also be specified for extension by the program. The *Buccaneer* pipeline runs multiple iterations of statistical chain tracing (identifying connected C^α^ atoms and docking sequence) followed by coordinate refinement using *REFMAC*. Currently, input maps should have a resolution of ∼5 Å or better. Users can adjust the overall sharpening level, and this can significantly improve the number of residues that are able to be correctly positioned. Models built by *Buccaneer* can be loaded into *Coot* for validation and further manual model building.

### 4.4. *Flex-EM*   

*Flex-EM* (Topf *et al.*, 2008 ▸; Joseph *et al.*, 2016 ▸) provides flexible fitting of rigid-body domains against EM volumes using real-space restraints. It requires an atomic model that has been rigid-body fitted and the corresponding map. Rigid-body domains must also be supplied, either defined manually or by using the *RIBFIND* task. *Flex-EM* is suitable for medium-resolution data sets, ∼15 Å or better, and two modes of refinement are available: conjugate-gradient minimization (CG) and simulated-annealing molecular dynamics (MD). By default, Ramachandran φ–ψ restraints are included, but these can be switched off as required. The CCF score (real-space cross-correlation) is recorded per iteration and is shown in the Results tab. *Flex-EM* is an extension of *Modeller* (Webb & Sali, 2016 ▸) and requires *Modeller* to be installed separately (https://www.salilab.org/modeller/).

### 4.5. *RIBFIND*   

*RIBFIND* was developed by Pandurangan & Topf (2012*a* ▸,*b* ▸) to cluster rigid-body domains for *Flex-EM*. *DSSP* (Kabsch & Sander, 1983 ▸) is used to identify secondary-structure elements (SSEs) *via* neighbourhood-based clustering. The number of SSEs generated by the clustering is tuneable *via* two spatial proximity parameters: the residue-contact distance, which represents the contact between any two residues (side-chain centroid–centroid distance) enforcing the minimum distance between the clustered SSEs, and the cluster cutoff, which clusters any two SSEs based on the percentage of residues in contact with them. The contact distance is set as an *a priori* input and the cluster cutoff can be tuned visually, post-processing, using a *JSmol* (Hanson *et al.*, 2013 ▸) plugin widget.

### 4.6. *REFMAC*   

*REFMAC* has been used extensively for high-resolution structure refinement of macromolecules against X-ray crystallographic data (Murshudov *et al.*, 2011 ▸). It has recently been re-optimized for use with high-resolution EM maps from SPRs (Brown *et al.*, 2015 ▸). *REFMAC* uses a maximum-likelihood target function to simultaneously optimize the agreement of the input model with the experimental density and with expected stereochemical restraints. Here, the experimental gradients are calculated in reciprocal space. This has several advantages compared with real-space refinement beyond the convenience of use for pre-existing MX-derived applications, which include the following: all parameters can be refined against all data, resolution-dependent weighting can be applied, and overall quality metrics, in particular Fourier shell correlation (FSC), become available (Brown *et al.*, 2015 ▸). Real-space refinement also has advantages such as local parameter optimization and rapid local adjustments (for example rotamer searches), and allows user visualization and intervention. Therefore, it is recommended that *REFMAC* is used in concert with the real-space refinement and validation routines available in *Coot*, and the *CCP-EM* GUI provides quick launching from the *REFMAC* task window for this.

For use with EM data, the *REFMAC* process itself requires some data manipulation. The *CCP-EM* task (Fig. 5 ▸) uses the pipeline functionality to handle this automatically for the convenience of the user. The input map (.mrc) is converted internally to structure factors (.mtz) and map sharpening can be applied at this point. If selected, the ‘find in map’ option will add a *MOLREP* process to the pipeline to perform rigid-body docking. The ‘local’ refinement mode extracts the map volume around the input model coordinates and refines against this volume only (as opposed to the whole volume). This is useful for very large systems or where atomic models are only available for specific domains; however, careful manipulation is required to place the refined model back into the correct orientation (with respect to the whole volume) and this is handled automatically after refinement in the *CCP-EM* pipeline. If the macromolecule includes nucleic acids, *LIBG* (Brown *et al.*, 2015 ▸) can be added to the pipeline to automatically generate additional restraints to maintain base pairing and stacking. Finally, if appropriate half-maps are available after three-dimensional map reconstruction, cross-validation processes, as described by Brown *et al.* (2015 ▸), are added to the pipeline and run automatically.

*REFMAC* has a number of other options which are helpful for EM refinement and are exposed in the *CCP-EM* task window. These include setting the relative weight of the experimental and stereochemical restraints (along with an option to automatically determine weights), pre-refinement map sharpening and the use of additional restraints, including jelly-body restraints or external restraints from *ProSMART*.

### 4.7. *ProSMART*   

*ProSMART* (*Procrustes Structural Matching Alignment and Restraint Tool*) was originally developed by Nicholls *et al.* (2012 ▸) to aid the modelling of low-resolution X-ray structures *via* the generation of additional restraints, and has since been successfully applied to model refinement from cryo-EM data (Brown *et al.*, 2015 ▸). The external restraints generated by *ProSMART* can be added to *REFMAC* to supplement its standard dictionary of restraints. The additional interatomic distances can be generated in a number of ways: from high-resolution homologous structures, secondary-structure restraints or multiple chains within the target model, or the modelled structure can be self-restrained to the current conformation. The *CCP-EM* task allows the generation of these restraints, which can then be visualized in *Coot* and edited as required. They can be then be added to the *REFMAC* process *via* the input in the ‘external restraints’ section of the setup task.

### 4.8. *Map to MTZ*   

This task runs the ‘sfcalc’ mode of *REFMAC* and has two specific roles: the conversion of real-space maps in MRC format to reciprocal-space structure factors in MTZ format, and map sharpening/blurring (Nicholls *et al.*, 2012 ▸). Presently, the optimum sharpening coefficient (where ‘optimum’ means maximizing the interpretable density features) cannot be analytically determined either locally or globally, although attempts are ongoing. Visual inspection of maps with different sharpening factors, however, reveal significant differences, which can impact both manual and automated model building. Therefore, the *Map to MTZ* *CCP-EM* task applies an array of sharpening factors for assessment. A Wilson plot is displayed, allowing inspection of potential truncation pathologies arising from over-sharpening (Nicholls *et al.*, 2012 ▸), and the task is linked to *Coot* for visual inspection. This process is recommended for new data sets and it should be noted that local areas may have different optimal sharpening values.

### 4.9. *TEMPy*   

*TEMPy* implements a wide variety of scoring functions for model-to-map and map-to-map fits (Vasishtan & Topf, 2011 ▸; Farabella *et al.*, 2015 ▸; Joseph *et al.*, 2017 ▸), as well as other functions for map and model manipulations. It was designed as a Python library with a series of command-line scripts for useful routines. The *CCP-EM* suite currently has two *TEMPy* interfaces: *TEMPy:DiffMap* and *TEMPy:SMOC*. *TEMPy:DiffMap* produces difference density maps *via* the scaled subtraction of two experimental maps or an experimental map and a coordinate model (where the model is converted into a calculated volume). *TEMPy:SMOC* (Segment-based Manders’ Overlap Coefficient) is a local validation metric that produces a correlation score per residue calculated on segments of overlapping residue windows. This method of calculating localized fit to density can alert users to areas of a model which require attention, and is particularly suited to medium-resolution data sets (∼4–10 Å).

### 4.10. MRC image-processing system   

The MRC–LMB have provided a comprehensive software library for EM since the early days (Crowther *et al.*, 1996 ▸), together with a large set of programs and utilities (for example *XIMDISP*; Smith, 1999 ▸). *CCP-EM* has taken over long-term maintenance of this software and it is distributed as part of the suite. The majority of the routines are written in Fortran and are available *via* a CLI; however, two programs (*mrc*2*tif* and *mrcallspacea*) have *CCP-EM* GUIs and more will be produced if requested by the community.

## 5. Availability and future plans   

Currently, *CCP-EM* binary installations are available to download from http://www.ccpem.ac.uk and are available for 64-bit Linux and Apple platforms, with plans to extend to 64-bit Windows in the near future. The Linux distributions are built on a nightly basis using a Jenkins CI (continuous integration) platform hosted by SESC Build Service (STFC). This autonomous system compiles the code, runs the unit tests and reports the status of the distribution, highlighting any potential errors that have inadvertently been introduced. This allows the rapid development and deployment of new functionality.

Here, we have described the current functionality of the *CCP-EM* software suite, which is focused on the fitting and building of atomic models, while also providing generic tools for manipulating and visualizing image and volume data. We are currently working on extending the range of applications for single-particle reconstruction, and have plans to cover subtomogram averaging as well. We are working closely with the EMDB (Patwardhan, 2017 ▸) on providing tools for map and model validation as part of structure determination, *i.e.* to be applied prior to deposition (Henderson *et al.*, 2012 ▸). Documentation on the *CCP-EM* software is available from the website, and further information, feedback and user questions can be obtained from the mailing list at http://www.jiscmail.ac.uk/CCPEM. Software developers who wish to discuss including their programs in the *CCP-EM* distribution should contact the authors of this article.
